# Uncertainty Analysis in Humidity Measurements by the Psychrometer Method

**DOI:** 10.3390/s17020368

**Published:** 2017-02-14

**Authors:** Jiunyuan Chen, Chiachung Chen

**Affiliations:** Department of Bio-industrial Mechatronics Engineering, National ChungHsing University, Taichung 40227, Taiwan; bse@dragon.nchu.edu.tw

**Keywords:** measurement uncertainty, psychrometer constant, dry bulb, wet bulb, humidity, regression analysis

## Abstract

The most common and cheap indirect technique to measure relative humidity is by using psychrometer based on a dry and a wet temperature sensor. In this study, the measurement uncertainty of relative humidity was evaluated by this indirect method with some empirical equations for calculating relative humidity. Among the six equations tested, the Penman equation had the best predictive ability for the dry bulb temperature range of 15–50 °C. At a fixed dry bulb temperature, an increase in the wet bulb depression increased the error. A new equation for the psychrometer constant was established by regression analysis. This equation can be computed by using a calculator. The average predictive error of relative humidity was <0.1% by this new equation. The measurement uncertainty of the relative humidity affected by the accuracy of dry and wet bulb temperature and the numeric values of measurement uncertainty were evaluated for various conditions. The uncertainty of wet bulb temperature was the main factor on the RH measurement uncertainty.

## 1. Introduction

Humidity is an important factor not only in various industries [1], but also in indoor environmental quality [2,3], in environment control processing [3], and in the assessment of heat stress, health and productivity for workers [4,5]. It affects evaporation and disease development in plants and the quality of food, chemicals and pharmaceuticals [1]. Accurate and reliable measurement of humidity is a key point for civil building [2,6] and health risks [7]. Typically, the amount of vapor contained in a moist air sample is expressed in terms of relative humidity (RH) [3].

Many sensors have been developed to measure RH. Popular commercial devices are chilled mirror hygrometers, electrical sensors and dry and wet bulb psychrometers [1,8]. The mirror hygrometer is the most accurate and it is commonly used for calibration of other instruments. Limitations of this equipment are its expense, sensitivity to contaminants and the requirement for skilled staff for its maintenance. Two types of electrical humidity sensors are resistive and capacitive sensors. Both types feature a fast response, good stability and little hysteresis [9]. The capacitive polymer sensor has a wider measurement range than the resistive type, however, the sensing elements of capacitive plates are exposed to condensation with high humidity and are then damaged. The main disadvantages of electrical sensors are that they are sensitive to contaminants, affected by ambient temperature and feature nonlinear calibration curves [9,10]. With careful calibration, the measurement uncertainty of these electrical sensors is >1.3% RH [11].

Because of the low cost and ease of use, psychrometry has been a popular method for measuring RH for a long time. This device involves a pair of electrical thermometers for measuring dry and wet bulb temperatures. The wet bulb thermometer is enclosed with a wick material that is maintained under wet conditions with distilled water. According to ISO standard 7726 [12], the wet thermometer should be ventilated at a sufficient velocity, generally at least 4 m/s to 5 m/s. In this way, the wet bulb temperature is close to the thermodynamic wet bulb temperature. The RH of air is then calculated by the dry and wet bulb temperature.

To ensure the accuracy of any humidity measurement, the factors affecting performance need to be considered. These factors include the accuracy of the two thermometers, the wind speed passing over the thermometers, the maintenance of the wetted condition for the wick materials surrounding the wet bulb thermometer, the care in shielding both sensors from radiation [13] and the selection of calculation equations. The choice of thermometers and maintenance of wet bulb conditions are the basic needs for measurements. However, the effect of calculation equations on RH needs to be studied. The measurement uncertainty of RH by the psychrometer method needs to be evaluated.

The calculation of RH by using dry and wet bulb temperature can be traced with thermodynamic theory. Theoretical formulas were proposed by ASHRAE [14,15]. These equations are derived from thermodynamic reasoning involving complex iterative calculation and require computer software for their calculation. Singh et al. [16] proposed a numerical calculation of psychrometric properties with a calculator, but the calculation of RH with dry and wet bulb temperature was still complex. Bahadori et al. [17] proposed a predictive tool to estimate RH using dry and wet bulb temperature that could be easily applied by an engineer without extensive mathematical ability. However, the equations still needed to be solved by iterative calculation.

Harrison and Wood [18] evaluated the effect of wind speed passing over the wet bulb temperature on the error sources of the humidity measurement and recommended that 2 m wind speeds should be >3 m/s. Ustymczuk and Giner [19] studied the effect of the performance of temperature sensors on the RH error and found that error increased linearly with increasing RH and decreased exponentially with increasing dry bulb temperature. The atmospheric pressure had only a slight effect on error. Mathioulakis et al. [20] demonstrated an evaluation method to calculate measurement uncertainties with indirect humidity measurement. Because of the strong non-linear characteristic of these calculation equations, the authors suggested using the Monte Carlo simulation for evaluation. Some empirical equations have been proposed to simplify the calculation of RH with dry and wet bulb temperature [21,22,23,24,25,26,27].

The RH value calculated with dry and wet bulb temperatures is called the indirect measurement. The difference between the actual and indirect measurement RH is defined as the error. Error is an idealized parameter, and the quantifying factors that affect it are difficult to determine. The measurement uncertainty was defined first in the ISO Guide [28]. The evaluation method has been described in detail [28,29,30]. According the ISO GUM, the measurement uncertainty was divided into A (by statistical method) and B type (by other information). The difference in error and uncertainty was defined clearly. The advantages of measurement uncertainty included the identification of the dispersion of results, estimated with a statistical method and quantification of the contribution of the uncertainty sources.

In this study, the predictive performance of these empirical equations was compared. The psychrometer coefficient of the empirical equation was calculated by an inverse technique. The relationship between this coefficient and dry and wet bulb temperatures was then established by regression analysis. The validity of the new empirical equation is reported. The ISO GUM concept was used to study the effect of the uncertainty of dry bulb temperature (T_d_) and wet bulb temperature (T_w_) on the measurement uncertainty of RH.

## 2. Theoretical Background

### Equations for Determining Psychrometric Constant

The empirical equation for calculating RH with dry and wet bulb temperatures is as follows:

P_w_ = P_ws_(T_w_) − A × P × (T_d_ − T_w_) (1)

where Pw is the partial pressure of water vapor in air in kPa, Pws(Tw) is the saturation vapor pressure of water at temperature Tw in kPa, Td is the dry bulb temperature in °C, Tw is the wet bulb temperature in °C, P is the standard atmosphere pressure in kPa, and A is the psychrometer coefficient in °C^−1^·kPa^−1^. The difference between T_d_ and T_w_ is called wet bulb depression.

RH is calculated as follows:

RH = P_w_/P_ws_ (T_d_) × 100% (2)

where RH is the relative humidity, and P_ws_ (T_d_) is the saturation vapor pressure of water at temperature T_d_ in kPa.

For calculating P_ws_, a simple equation was used with a the range of 0–100 °C [14]:

(3) $$P_{\mathrm{ws}}=0.61078\times \mathrm{Exp}[\frac{17.2694T}{T\;+\;237.3}]$$

where T is the air temperature in °C.

The standard atmosphere pressure P is considered in this study [6]:

P = 101.325 kPa
(4)

Equation (1) then could be expressed as follows:

P_w_ = P_ws_ (T_w_) − A_s_ × (T_d_ − T_w_) (5)

where A_s_ is the psychrometer constant in standard atmosphere pressure in °C^−1^.

Some empirical equations have been proposed [21,22,23,24,25,26,27]. The Sensiron recommended the A_s_ value in the range of 6.4 × 10^−4^ to 6.8 × 10^−6^ °C^−1^ [12,21]. Other equations are listed as Table 1.

To evaluate the predictive performance of the above equations, the predictive error of empirical equations is defined as follows:

E = RH_sta_ − RH_cal_ (6)

where E is the predictive error of the empirical equation in a percentage, RH_sta_ is the RH value calculated from the ASHRAE formula, and RH_cal_ is the RH value calculated from these empirical equations.

Besides the minimum and maximum E values, E_max_ and E_min_, a statistic |E|_ave_ is defined as a criterion for evaluating the predictive ability:

(7) $${\left|E\right|}_{\mathrm{ave}}=\left|E\right|/n$$

where |E| is the absolute value of E, and n is the number of data.

To establish the new RH_cal_ equation, the psychrometer efficient at standard atmosphere is defined as A_s_. A_s_ was determined by rearranging Equations (1) and (2) as follows:

(8) $$A_{s}=\frac{\mathrm{Pws}\left(T_{w}\right)\;-P_{W}}{T_{d}-T_{\omega }}$$

The calculation of A_s_ by Equation (8) was called the inverse technique. The evaluation of the measurement uncertainty is listed in Appendix A.

## 3. Materials and Methods

### 3.1. Equipment

**Figure 1:**
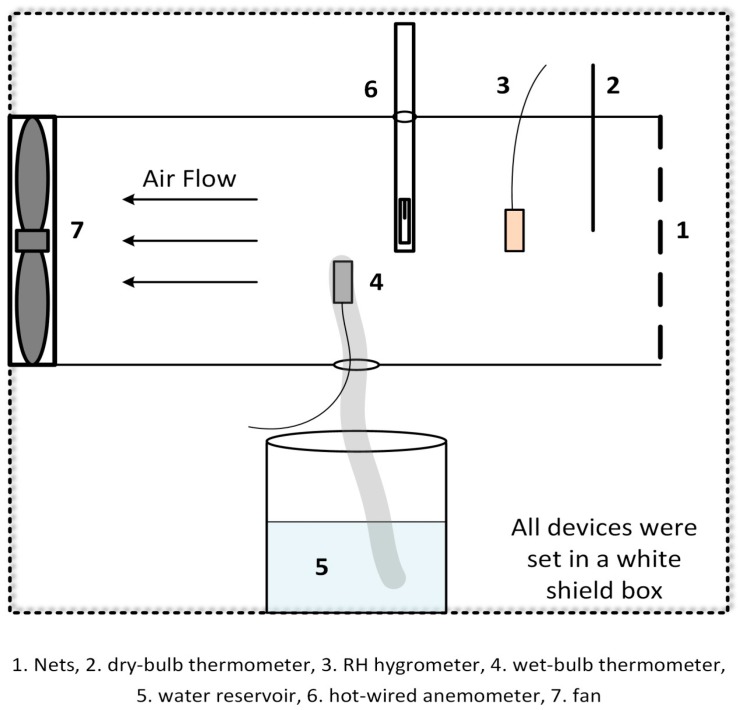
Experimental setup used for measurements (the figure is not to real scale).

The effect of air velocity on the measurement of wet bulb temperature was used as an example. The schematic of the experimental device is shown in Figure 1. The air was sucked into a wind tunnel by use of an adjustable fan. Two thermometers were used to measure the dry and wet bulb temperatures. The wet condition of the wet bulb thermometer was maintained with a wick and water reservoir. A resistant hygrometer served as the standard for RH measurement.

The air velocities were measured at several points to ensure the flow turbulence. Nets were used to filter particles and favoring the turbulence. The air velocity was adjusted by adjusting the fan speed. Air velocity was measured near the wet bulb thermometer by a hot-wired anemometer. A cotton wick 5 cm in length was attached to the wet bulb thermometer to maintain sufficient water to cool the sensor during aspiration. The measuring box was regularly maintained for each test. The wick must be clean and the de-ionized water was used as reservoir.

### 3.2. Sensors

The temperature was measured with use of the Sentron D9 temperature transmitter (Sentron Co., Taipei, Taiwan). This transmitter contains a Pt100 element. The error of this thermometer was 0.15 °C after calibration.

The RH was measured using a THT-B121 resistive transmitter (Shinyei Kalsha, Tokyo, Japan). The error of this RH sensor was 0.5% RH after calibration with several saturated salt solutions.

The air velocity passing over the wet-bulb thermometer was detected by use of the KANOMAX Hot-wired 6004 Anemometer (Kanomax USA, Andover, NJ, USA). The error was ±5% according to the manufacturer’s specifications.

### 3.3. Experimental Method

The experiment was performed in the laboratory. During the test, the air velocity was adjusted from 0 to 5 m/s. At each air velocity, the reading values of T_d_, T_w_, RH and wind velocity were recorded by use of a data logger (Delta-T, Cambridge, UK). The sampling frequency was 1 s until the reading values of T_w_ were stable. There were three measurements for each wind velocity. The actual T_w_ value was calculated with the measurement values of T_d_ and RH.

## 4. Results

### 4.1. Effect of Air Velocity on T_w_

**Figure 2:**
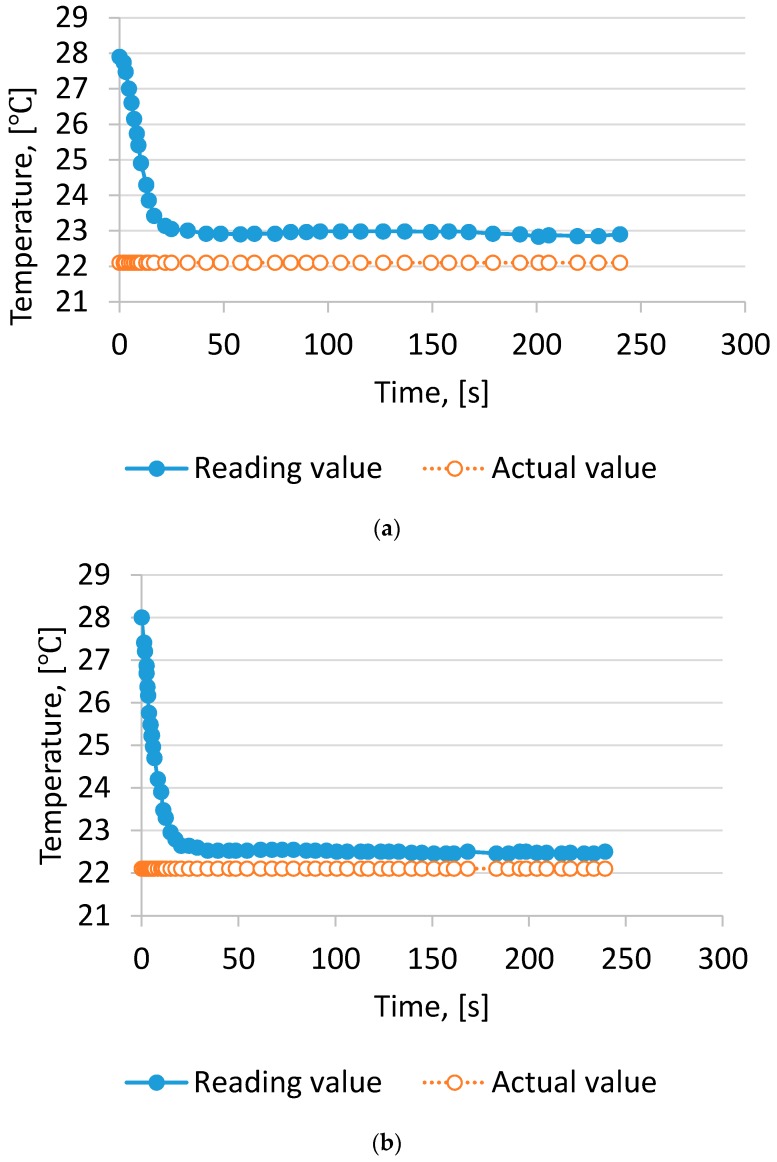

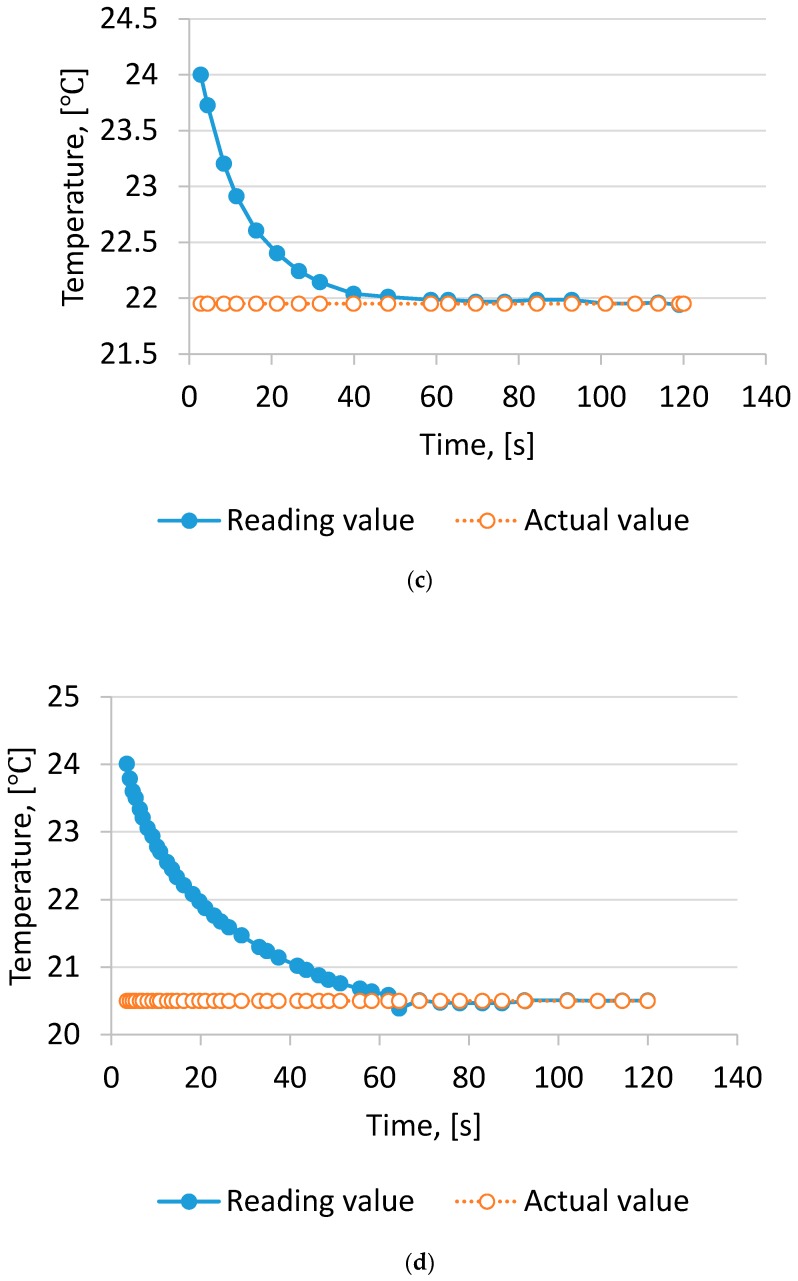

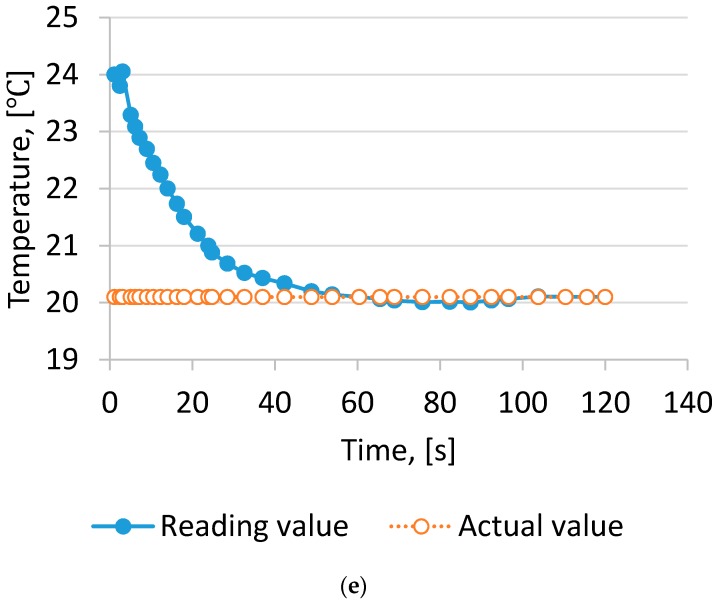
Wet bulb temperature readings with time. (**a**) wind velocity 1 m/s; (**b**) wind velocity 2 m/s; (**c**) wind velocity 3 m/s; (**d**) wind velocity 4 m/s; (**e**) wind velocity 5 m/s.

The effect of the air velocity on T_w_ measurement is shown in Figure 2. With 1.0 m/s, the deviation between reading values and actual values calculated by RH measurement of T_w_ was close to 0.8 °C.

The result could be explained by the lower air velocity passing the wet-bulb thermometer, the difference being due to the fact that Tw is not a thermodynamic quantity but only an indicator of the thermodynamic wet bulb temperature [15]. On increasing the air velocity to 2.0 m/s, the difference ranged from 0.3 °C to 0.4 °C. If the air velocity was >3.0 m/s, the measurement T_w_ was close to the actual value. The result was similar to findings by Harrison and Wood [18].

The error sources of the T_w_ measurement include the thermometer performance and the velocity of the air passing the wet bulb thermometer. The combined errors in this study ranged from 0.15 °C to 0.9 °C. If other factors were involved, the errors of T_w_ measurement may range from 0.2 °C to 1.0 °C. According to the study of Barber and Gu [31] ±0.5 °C was a common error for an aspirated psychrometer.

### 4.2. Comparison of Predictive Performance of Six Empirical Equations

The criteria for comparing the predictive performance of six empirical equations are given in Table 2. The |E|_ave_ was used to evaluate predictive performance. E_max_ and E_min_ show range of errors. From numerical values in Table 2, the Neiva et al. equation had the largest values for E_max_, E_min_ and |E|_ave_., so it was not adequate for RH calculation.

A_s_ values for three equations, Penman, BUT and Goff-Cratch, were constant. The criteria for predictive errors was higher for the Goff-Cratch equation than for the Penman and BUT equations. In the case of the Penman and BUT equations, the A_s_ value was 0.664 and 0.666 °C^−1^, respectively. Numeric values for A_s_ for the two equations were close. However, the criteria for predictive performance differed. The Penman equation had better performance than the BUT equation. The result indicated the sensitivity of the A_s_ value for the predictive performance of RH equations.

The A_s_ for three equations, Harrison, WMO and Neiva et al., all involved a linear relationship with T_w_ value. E_min_ was larger for the WMO than the Harrison equation for T_d_ < 40 °C, and |E|_ave_ values were smaller for the WMO than the Harrison equation for all 10 dry bulb temperatures. The Penman equation had the smallest values for E_min_, E_max_ and |E|_ave_. Therefore, the Penman equation had the best predictive performance among the six empirical equations.

**Figure 3:**
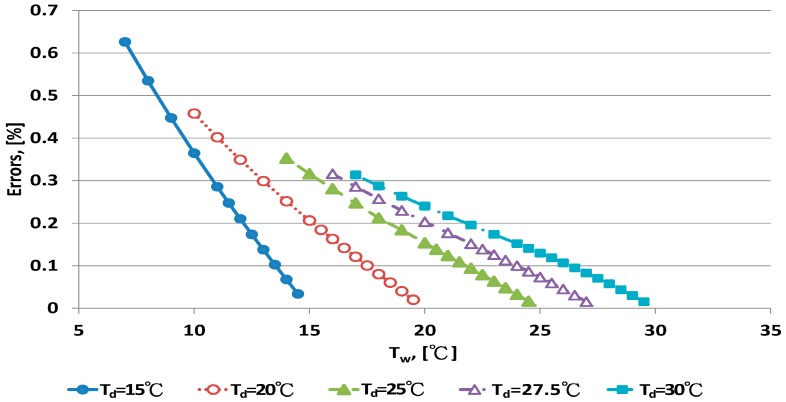
Error distribution of the Penman equation for dry bulb temperature <30 °C.

**Figure 4:**
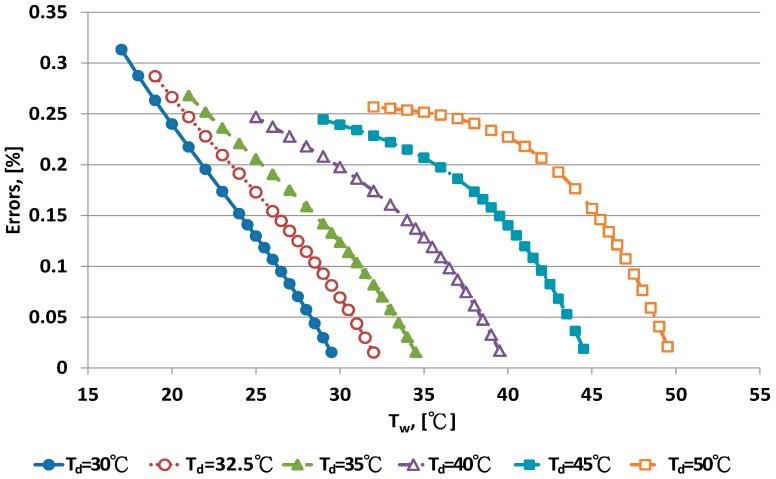
Error distribution of the Penman equation for dry bulb temperature >30 °C.

The error distribution of the Penman equation with 10 dry bulb temperatures is shown in Figure 3 and Figure 4. Error increased with decreasing wet bulb temperature at fixed dry bulb temperature. The data distribution of errors was curved. When the wet bulb temperature was close to the dry bulb temperature, that is, when RH increased to saturation, the predicted error decreased.

**Figure 5:**
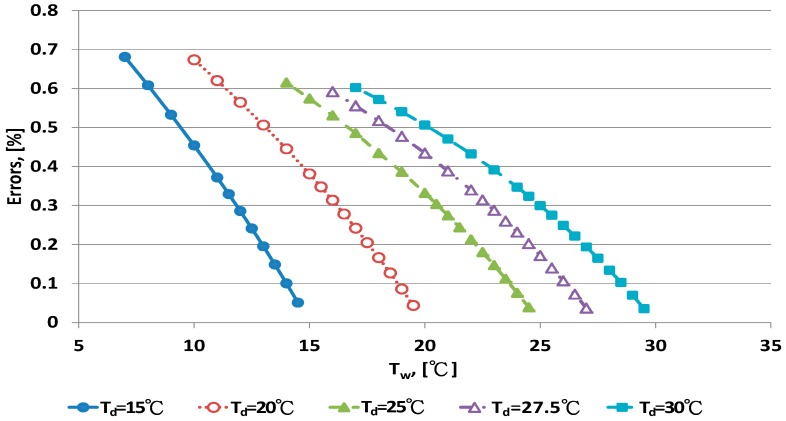
Error distribution of the WMO equation for dry bulb temperature <30 °C.

**Figure 6:**
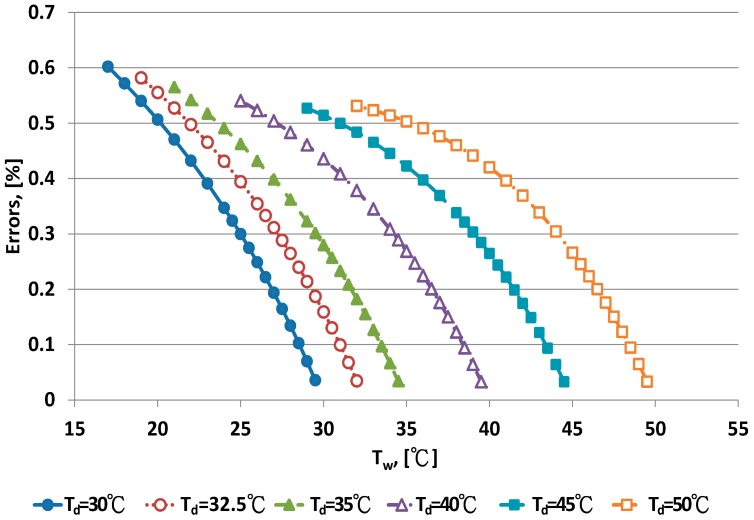
Error distribution of the WMO equation for dry bulb temperature >30 °C.

The Penman equation had better predictive ability at high than low RH. The limitation of electrical sensors is poor performance with high RH. The psychrometer method can be used for high RH measurement. The error distribution for the WMO equations under dry bulb temperatures is shown in Figure 5 and Figure 6. When the wet bulb temperature was near the dry bulb temperature, errors decreased. The error distribution patterns were similar to those for the Penman equation.

### 4.3. Development of a New A_s_ Equation

**Figure 7:**
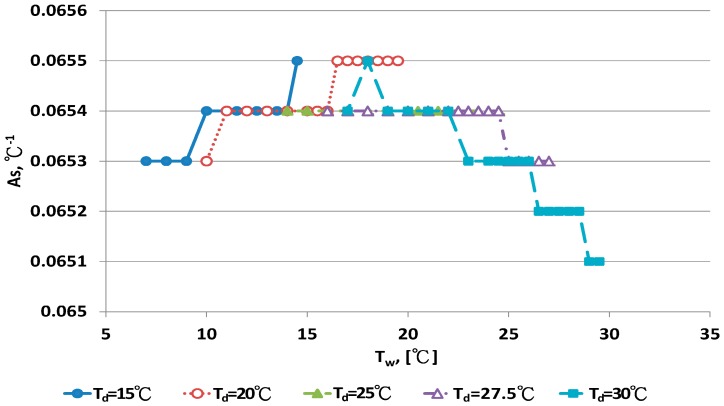
The relationship between A_s_ and wet-bulb temperatures with dry bulb temperature <30 °C.

**Figure 8:**
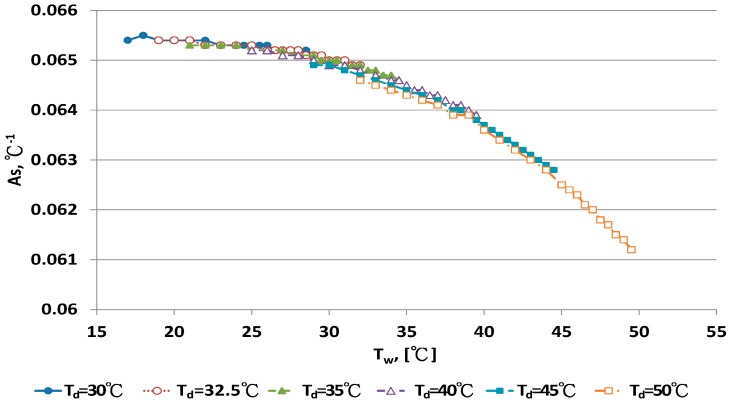
The relationship between A_s_ and wet-bulb temperatures with dry bulb temperature >30 °C.

A_s_ values at fixed dry bulb temperature and different wet bulb temperature were calculated by the inverse technique from Equation (14). The relationship between A_s_ and wet bulb temperatures under 10 dry bulb temperatures is illustrated in Figure 7 and Figure 8. Figure 7 shows that A_s_ was nearly constant for dry bulb temperatures <30 °C. A_s_ was close to 0.0654 °C^−1^. The data distribution for A_s_ with T_d_ > 30 °C in Figure 7 presents a clear curve shape. A_s_ strongly depended on the wet bulb temperature, T_w_, and weakly on dry bulb temperature, T_d_. The relationship for A_s_ and the two temperatures were evaluated by regression analysis:

A_s_ = 0.0654 °C^−^^1^, T_d_ < 30 °C (9)

A_s_ = 0.0637485 + 0.000187508 T_w_ − 4.376670 × 10^−6^ T_w_^2^ − 1.21851 × 10^−5^ T_d_ R^2^ = 0.99483, s = 4.73762 × 10^−5^, T_d_ > 30 °C, (10)

The new A_s_ equation was incorporated into Equation (1). Predictive errors for this new A_s_ equation are in Table 1. Three criteria, E_min_, E_max_ and |E|_ave_, were lower for the new A_s_ equation than other empirical equations. At T_d_ = 15 °C, |E|_ave_ for the new A_s_, Penman and WMO equations was 0.0988%, 0.2261% and 0.3331%, respectively. At T_d_ = 30 °C, |E|_ave_ for the above three equations was 0.0058%, 0.1422% and 0.3016%, respectively. At T_d_ = 50 °C, |E|_ave_ for the above three equations was 0.00458%, 0.1719% and 0.3190%, respectively. The predictive errors of the new A_s_ equation improved significantly.

**Figure 9:**
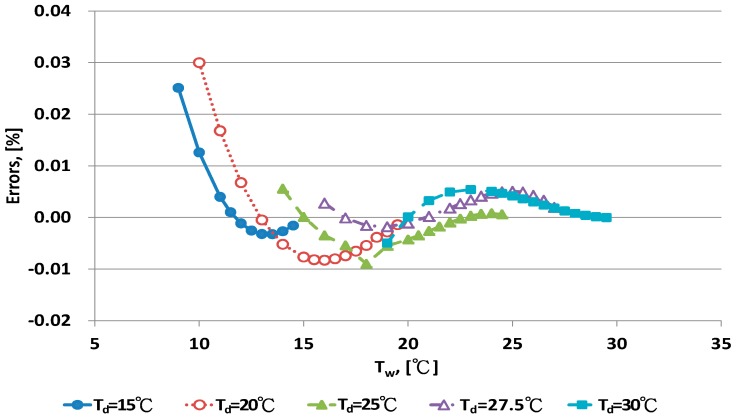
Error distribution of the new A_s_ equation with dry bulb temperature <30 °C.

**Figure 10:**
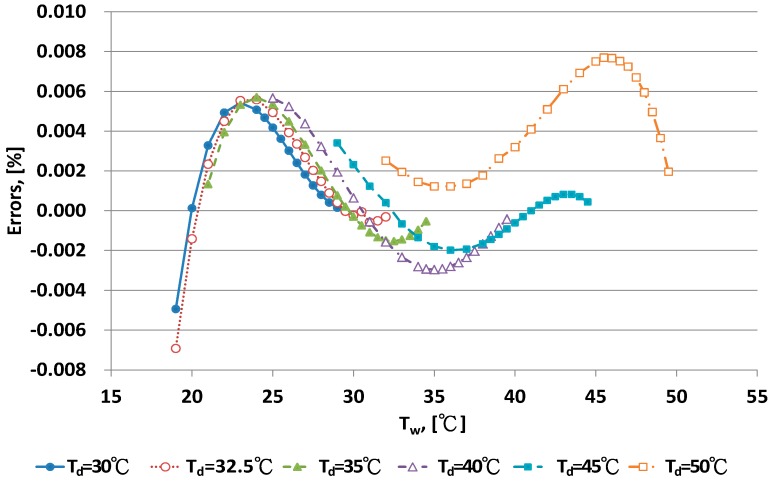
Error distribution of the new A_s_ equation with dry bulb temperature >30 °C.

The error distribution for the new A_s_ equation for different dry bulb temperatures is shown in Figure 9 and Figure 10. With T_d_ < 25 °C, larger errors were found at the lower range of T_w_. With T_d_ > 30 °C, error distributions were curved. With a more complex form of the A_s_ model, for example, when higher order polynomial equations were used, the error distribution of the curve shapes could be improved. However, the new A_s_ equation, Equation (10), could be easily computed with a calculator. The predictive value of errors was <0.1% RH. This error could be acceptable in term of practical application for humidity measurement [1,8], so Equation (10) is recommended as the adequate A_s_ equation.

Simoes-Moreire found that most of the empirical equations for the psychrometer coefficient of A were presented in a fixed range or as a constant [32]. Some researchers have proposed a linear relationship for the A value and wet bulb temperature [25,26,27]. However, the linear T_w_ model for A_s_ did not improve the predicted values of RH. The new A_s_ model proposed in this study can significantly improve the predictive ability for RH measurement.

### 4.4. Measurement Uncertainty of Humidity Calculated by T_d_ and T_w_ Values

The measurement uncertainty of humidity of a direct method has been investigated [11]. The evaluation of measurement uncertainty was studied with the new A_s_ equation developed in this study.

The typical uncertainty of a dry bulb thermometer, u(T_d_), carefully calibrated was 0.15 °C [33]. The estimated uncertainty of a wet bulb thermometer, u(T_w_), ranged from 0.15 °C to 1.0 °C.

**Figure 11:**
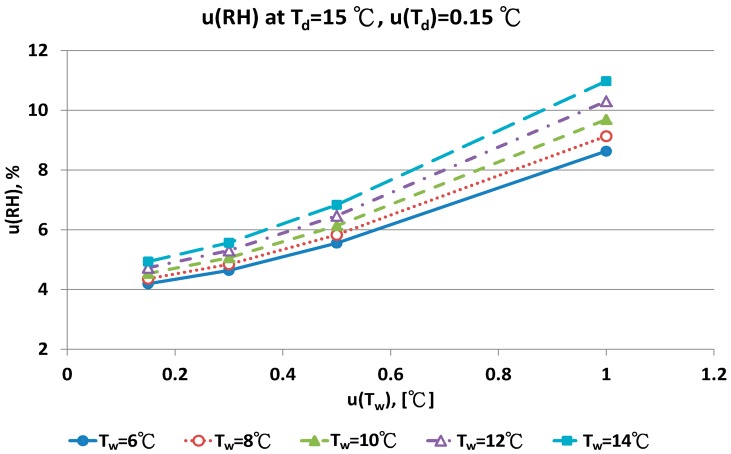
Uncertainties of relative humidity calculated with Equation (9) at T_d_ = 15 °C, u(T_d_) = 0.15 °C, T_w_ = 6~14 °C and u(T_w_) = 0.1~1 °C.

**Figure 12:**
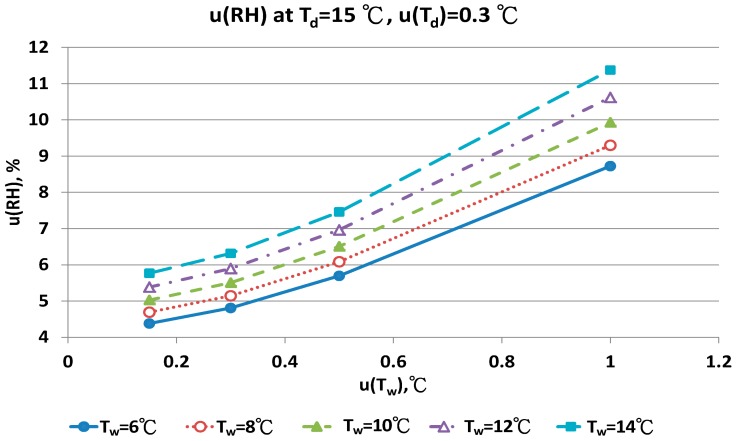
Uncertainties of relative humidity calculated with Equation (9) at T_d_ = 15 °C, u(T_d_) = 0.30 °C, T_w_ = 6~14 °C and u(T_w_) = 0.1~1 °C.

**Figure 13:**
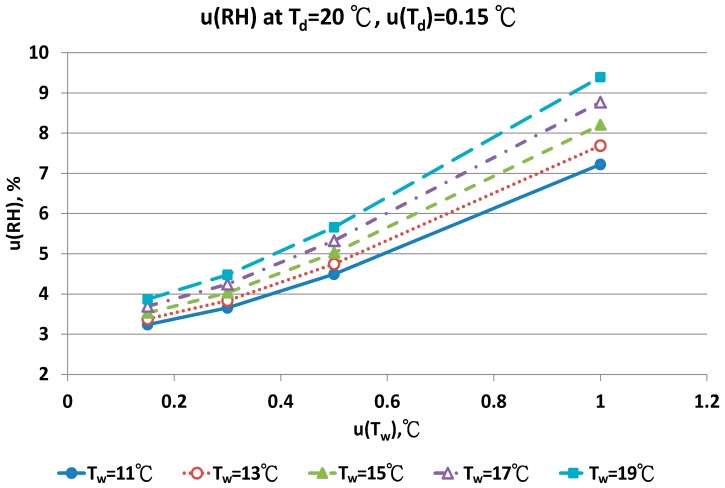
Uncertainties of relative humidity calculated with Equation (10) at T_d_ = 20 °C, u(T_d_) = 0.15 °C, T_w_ = 11~19 °C and u(T_w_) = 0.1~1 °C.

**Figure 14:**
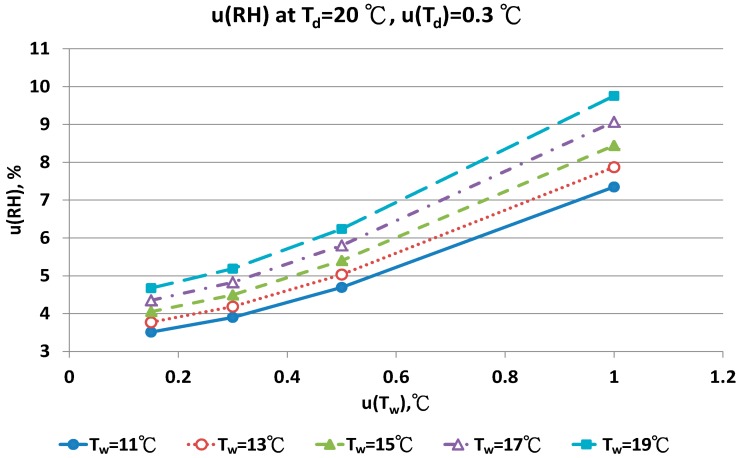
Uncertainties of relative humidity calculated with Equation (10) at T_d_ = 20 °C, u(T_d_) = 0.3 °C, T_w_ = 11~19 °C and u(T_w_) = 0.1~1 °C.

**Figure 15:**
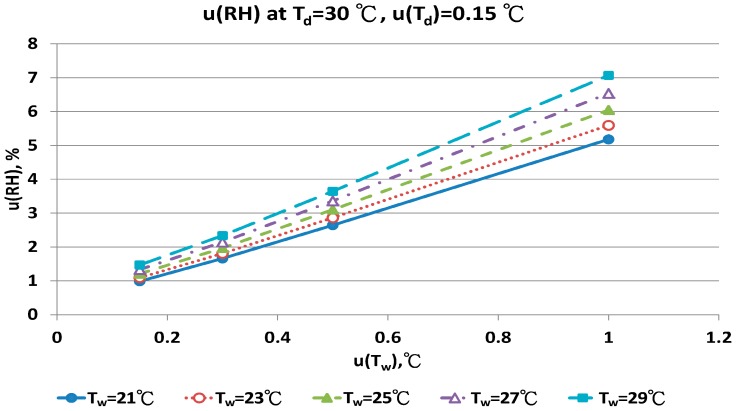
Uncertainties of relative humidity calculated with Equation (10) at T_d_ = 30 °C, u(T_d_) = 0.15 °C, T_w_ = 21~29 °C and u(T_w_) = 0.1~1 °C.

**Figure 16:**
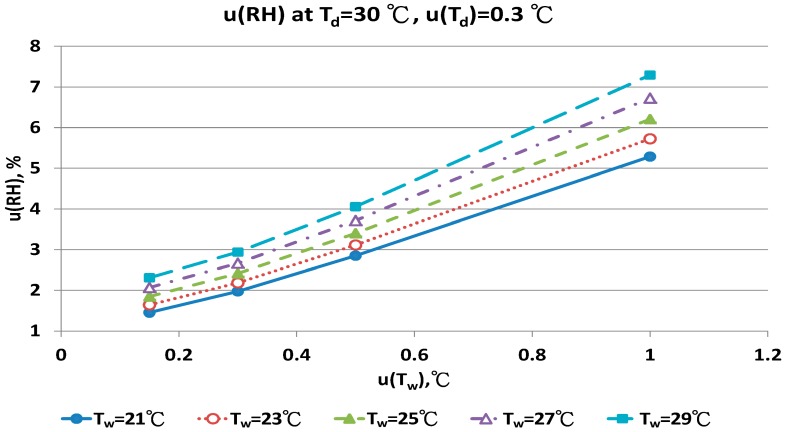
Uncertainties of relative humidity calculated with Equation (10) at T_d_ = 30 °C, u(T_d_) = 0.3 °C, T_w_ = 21~29 °C and u(T_w_) = 0.1~1 °C.

**Figure 17:**
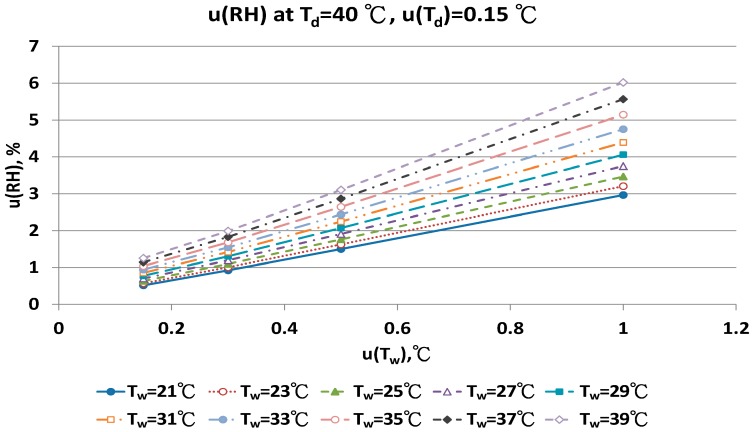
Uncertainties of relative humidity calculated with Equation (10) at T_d_ = 40 °C, u(T_d_) = 0.15 °C, T_w_ = 21~39 °C and u(T_w_) = 0.1~1 °C.

**Figure 18:**
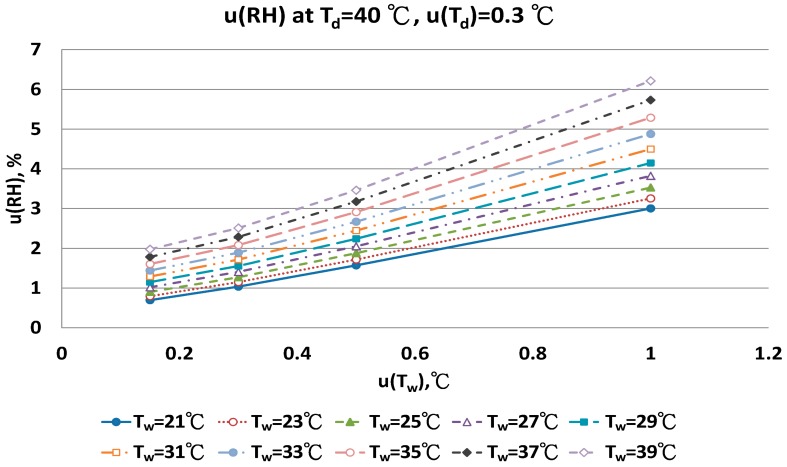
Uncertainties of relative humidity calculated with Equation (10) at T_d_ = 40 °C, u(T_d_) = 0.3 °C, T_w_ = 21~39 °C and u(T_w_) = 0.1~1 °C.

The combined uncertainty of the RH value, u(RH), evaluated by Equations (A5)–(A12) for four dry bulb temperatures with two uncertainties u(T_d_), 0.15 °C and 0.3 °C in different wet bulb temperatures, is in Figure 11, Figure 12, Figure 13, Figure 14, Figure 15, Figure 16, Figure 17 and Figure 18. The results of the calculation of u(RH) with other u(T_d_), 0.1 and 0.5, are available in supplemental information.

Figure 11 show that with increased uncertainty of the wet bulb thermometer, u(T_w_) enhanced the combined uncertainties of u(RH). At the same u(T_w_), higher wet bulb temperature induced larger u(RH) value.

With the smallest u(T_w_) value, 0.1 °C, the combined uncertainty of u(RH) was 4.35% and 4.93% for the T_w_ at 6 °C and 14 °C, respectively. If the u(T_w_) value was 0.5 °C, the u(RH) was 5.83% and 6.83% for the T_w_ at 6 °C and 14 °C, respectively. The uncertainty of u(T_w_) affected the u(RH) value significantly. With the largest u(T_w_), 1.0 °C, the largest uncertainty was 8.63% and 10.98%, respectively.

The contributions of u(T_w_) are the performance of the sensor and the measurement technique for the wet bulb condition. If the uncertainty of wet bulb temperature was >0.5 °C, the u(RH) ranged from 5% to 11%. The measurement error of RH with the psychrometer was obvious. A similar result could be found for u(T_d_) = 0.3 °C. At the dry bulb temperature of 20 °C, the distribution of u(RH) in different wet bulb temperatures, u(T_d_) and u(T_w_) values was similar to the results at 15 °C (Figure 13 and Figure 14). However, the numeric values of u(RH) were lower than the results at 15 °C. If the u(T_d_) and u(T_w_) was 0.15 °C, the u(RH) was 3.24% and 3.87%, respectively. With the u(T_d_) = 0.15 °C and u(T_w_) = 0.5 °C, the u(RH) ranged from 4.49% to 5.16% for different T_w_ values. At the worst conditions of u(T_w_) = 1.0 °C, the u(RH) values ranged from 7.22% to 9.40%.

The u(RH) values for the T_d_ at 30 °C in different T_w_, u(T_d_) and u(T_w_) values are in Figure 15 and Figure 16. With u(T_d_) and u(T_w_) = 0.15 °C, the results for u(RH) ranged from 0.99% to 1.47%. With u(T_w_) = 0.5 °C, the u(RH) ranged from 2.64% to 3.64%. At the worst conditions of u(T_w_) = 1.0 °C, the u(RH) ranged from 5.18% to 7.07%. The RH values calculated by the new psychrometric equation with high dry bulb temperature had smaller u(RH) values.

The distribution of u(RH) of 40 °C T_d_ in different u(T_d_), u(T_w_) and T_w_ conditions are in Figure 17 and Figure 18. Nine T_w_ were considered. With u(T_d_) = 0.15 °C, the u(RH) ranged from 1.63% to 3.11% with u(T_w_) = 0.5 °C, and from 2.97% to 6.02% with u(T_w_) = 1.0 °C.

The u(RH) values at higher T_d_, 30 °C and 40 °C, were less than at T_d_ 15 °C and 20 °C. The result confirmed that the new psychrometric A_s_ equation was adequate for RH measurement in high temperature.

## 5. Conclusions

This work investigated some empirical equations for calculating the relative humidity (RH) from indirect measurement of dry and aspirated wet bulb temperatures. The standard value for RH was obtained from the equations reported in the ASHRAE Handbook. The Penman equation had the best predictive ability among the six equations tested with dry bulb temperature ranging from 15 °C to 50 °C. Some equations with a linear relationship of psychrometer coefficients and wet bulb temperature did not have good predictive ability. At a fixed dry bulb temperature, the increase in wet bulb depression increased the errors. The psychrometer method is adequate for measuring high RH.

A relationship between the psychrometer constant A_s_ and dry and wet bulb temperature was established by regression analysis. The new A_s_ equation included the polynomial from of T_w_. With this new A_s_ equation, the average predictive error was <0.1% RH.

The measurement uncertainty of RH calculated from dry and wet bulb temperature with this new A_s_ equation was evaluated. At the T_d_ values of 10 °C and 25 °C, the combined uncertainty of RH values ranged from 3.2% to 4.0% with u(T_w_) = 0.15 °C, 4.69% to 7.46% with u(T_w_) = 0.5 °C and 6.5% to 11.0% with u(T_w_) = 1.0 °C. At the T_d_ values of 30 °C and 40 °C, the combined uncertainty of RH values ranged from 0.52% to 2.31% with u(T_w_) = 0.15 °C, 1.72% to 4.06% with u(T_w_) = 0.5 °C and 2.97% to 7.29% with u(T_w_) = 1.0 °C.

The uncertainty of T_w_ had a significant effect on the combined uncertainty of RH. The uncertainty sources of T_w_ were performance of the thermometer and maintenance of wet bulb conditions. A quantification method was provided to evaluate measurement errors in calculating RH with dry and wet bulb temperatures.

## Figures and Tables

**Table 1:** Empirical equations used in this study.

1	Penman equation [22]
	P_w_ = P_ws_ (T_w_) − 0.0664 × (T_d_ – T_w_)
2	Goft-Cratch equation [23]
	P_w_ = P_ws_ (T_w_) − 0.067193 × (T_d_ – T_w_)
3	British United Turkeys (BUT) equation [24]
	P_w_ = P_ws_ (T_w_) − 0.066 × (T_d_ – T_w_)
4	Harrison equation [25]
	P_w_ = P_ws_ (T_w_) − 0.067 × (1 + 0.00115T_w_) × (T_d_ – T_w_)
5	World meteorological Organisation (WMO) equation [26]
	P_w_ = P_ws_ (T_w_) − 0.0662795 × (1 + 0.000944T_w_) × (T_d_ – T_w_)
6	Nevia et al. equation [27]
	P_w_ = P_ws_ (T_w_) − 0.0647164 × (1 + 0.00504T_w_) × (T_d_ – T_w_)

**Table 2:** Criteria for the evaluating six empirical equations for calculating relative humidity.

Temp. (°C)	Criteria	A_s_ (This Study) °C^−1^	Penman	BUT	Goff-Cratch	Harrison	WMO	Neiva et al.
15	Emin	−0.0032	0.0278	0.0336	0.0510	0.2783	0.0508	0.1171
	Emax	0.0476	0.5321	0.6259	0.1041	0.9483	0.6808	0.8323
	|E|_ave_	0.0988	0.2261	0.2691	0.3966	0.6492	0.3331	0.5575
20	Emin	−0.00823	0.1999	0.0115	0.0370	0.2353	0.0435	0.1200
	Emax	0.0300	0.4577	0.2866	0.7969	0.9548	0.6735	1.3476
	|E|_ave_	0.0079	0.1917	0.1947	0.3443	0.6330	0.3332	0.7268
25	Emin	−0.0090	0.0164	0.0195	0.0289	0.0557	0.0387	0.1161
	Emax	0.0056	0.3528	0.4223	0.6282	0.9358	0.6150	1.3540
	|E|_ave_	0.0448	0.1531	0.1841	0.2759	0.4612	0.3091	0.8033
27.5	Emin	−0.0018	0.0156	0.0183	0.0264	0.2815	0.0370	0.1126
	Emax	0.0051	0.3161	0.3187	0.5645	0.9617	0.5918	1.4235
	|E|_ave_	0.0029	0.1418	0.1696	0.2523	0.6542	0.3016	0.8282
30	Emin	−0.0423	0.0153	0.0176	0.0246	0.2474	0.0356	0.1080
	Emax	0.0054	0.3132	0.3745	0.5562	0.9635	0.6021	1.4964
	|E|_ave_	0.0058	0.1422	0.1693	0.2494	0.6368	0.3205	0.8774
32.5	Emin	−0.0069	0.0153	0.0174	0.0234	0.2603	0.0346	0.1048
	Emax	0.0056	0.2870	0.3423	0.5060	0.9651	0.5818	1.5331
	|E|_ave_	0.0024	0.1384	0.1628	0.2349	0.6418	0.3068	0.8004
35	Emin	−0.0015	0.0156	0.0174	0.0267	0.2715	0.0338	0.1008
	Emax	0.0057	0.2679	0.3119	0.4654	0.9665	0.5649	1.5543
	|E|_ave_	0.00216	0.1576	0.1576	0.2224	0.6474	0.3015	0.8851
40	Emin	−0.0030	0.0170	0.0183	0.0223	0.0411	0.0329	0.0929
	Emax	0.0057	0.2470	0.2877	0.4083	0.7609	0.5406	1.5631
	|E|_ave_	0.0024	0.1388	0.1566	0.2095	0.4017	0.3015	0.8751
45	Emin	−0.0020	0.0188	0.0199	0.0230	0.0399	0.0327	0.0858
	Emax	0.0034	0.2444	0.2778	0.3768	0.7177	0.5271	1.5428
	|E|_ave_	0.0011	0.1511	0.1656	0.2088	0.3910	0.3016	0.8592
50	Emin	−0.0076	0.0210	0.0218	0.0242	0.0389	0.0331	0.0797
	Emax	0.0077	0.2568	0.2859	0.3725	0.7041	0.5313	1.5342
	|E|_ave_	0.0046	0.1719	0.1848	0.2232	0.4015	0.3190	0.8706

## Supplementary Materials

The following are available online at http://www.mdpi.com/1424-8220/17/2/368/s1, Figures S1–S8.

## Nomenclature

A	psychrometric coefficient °C^−1^·kPa^−1^
A_s_	psychrometric constant incorporated air atmosphere term, °C^−1^
e	vapor pressure, kPa
e_s_	saturated vapor pressure, kPa
E	error of predictive performance, %
E_max_	maximum error of predictive performance, %
E_min_	minima error of predictive performance, %
|E|	absolute error of predictive performance, %
|E|_ave_	average of |E|
n	number of data
P	atmosphere air pressure, kPa
RH_cal_	calculated RH value from empirical equation
RH_sta_	standard RH value from ASHRAE Handbook
T	temperature of air, °C
T_d_	dry bulb temperature, °C
T_w_	wet bulb temperature, °C
u(RH)	uncertainty of relative humidity
u(T_d_)	uncertainty of dry bulb temperature
u(T_w_)	uncertainty of wet bulb temperature

## Appendix A. Evaluation of the Measurement Uncertainty

The steps to evaluate uncertainty are as follows [28,29,30]:

1 Model the measurement
  *y* is not measured directly and is determined from K quantities $x_{1},x_{2},......x_{k}$,
  The functional relationship is as follows:
  (A1) $$y=f(x_{1},x_{2},......x_{k})$$
2 Ensure the uncertainty source $x_{i}$ and calculate the estimated values of $x_{i}$
  (A2) $${u_{c}}^{2}[y]=\sum_{i=1}^{n}{\left[\frac{\partial y}{\partial x_{i}}\right]}^{2}u^{2}(x_{i})$$
  where $u_{c}(y)$ is the combined uncertainty and *y* is the output quantity.
3 Evaluate the uncertainty classified as A and B types.
4 Estimate the covariance of each $x_{i}$.
5 Calculate the sensitivity coefficient, $c_{i}$
  (A3) ci=∂f∂xi
6 Calculate the combining uncertainty and effective degree of freedom.
7 Determine a coverage factor and expanded uncertainty.
8 Report the uncertainty.
  The uncertainty of RH value that calculated from T_d_ and T_w_ values.
  (A4) $$\mathrm{RH}=\frac{P_{w}}{P_{ws}\left(T_{d}\right)}=\frac{P_{ws}\left(T_{w}\right)-A_{s}\left(T_{d}-T_{w}\right)}{P_{ws}\left(T_{d}\right)}$$
  By Equation (A2), the uncertainty of RH can be calculated follows:
  (A5) $${u_{c}}^{2}[RH]={\left(\frac{\partial RH}{\partial T_{d}}\right)}^{2}u^{2}(T_{d})+{\left(\frac{\partial RH}{\partial T_{w}}\right)}^{2}u^{2}(T_{w})$$
  (A6) $$\frac{\partial P_{ws}\left(T_{d}\right)}{\partial T_{d}}=2502.99\;\exp\left(\frac{17.2694{\;T}_{d}}{T_{d}+237.3}\right){\left(\frac{1}{T_{d}+237.3}\right)}^{2}$$
  (A7) $$\frac{\partial P_{ws}\left(T_{w}\right)}{\partial T_{w}}=2502.99\;\exp\left(\frac{17.2694{\;T}_{w}}{T_{w}+237.3}\right){\left(\frac{1}{T_{w}+237.3}\right)}^{2}$$
  If A_s_ is a constant,
  (A8) $$\frac{\partial \mathrm{RH}}{\partial T_{w}}=\frac{1}{p_{ws}\left(T_{d}\right)}\left[\frac{\partial p_{ws}\left(T_{w}\right)}{\partial T_{w}}+A_{s}\right]\;=\frac{1}{P_{ws}\left(T_{d}\right)}\left[\frac{\partial P_{ws}\left(T_{w}\right)}{\partial T_{w}}+A_{s}\right]$$
  (A9) $$\frac{\partial \mathrm{RH}}{\partial T_{d}}=\frac{1}{{\left[P_{ws}\left(T_{d}\right)\right]}^{2}}\left[-A_{s}\cdot p_{ws}\left(T_{d}\right)-\left[p_{ws}\left(T_{w}\right)-A_{s}\left(T_{d}-T_{w}\right)\right]\frac{\partial P_{ws}\left(T_{d}\right)}{\partial T_{d}}\right]$$
  If $A_{s}=C_{0}+C_{1}T_{w}+C_{2}{T_{w}}^{2}+C_{3}T_{d}$,
  (A10) $$\mathrm{RH}=\frac{1}{P_{ws}\left(T_{d}\right)}\left[p_{ws}\left(T_{w}\right)-\left(C_{0}+C_{1}T_{w}+C_{2}{T_{w}}^{2}+C_{3}T_{d}\right)\left(T_{d}-T_{w}\right)\right]$$
  (A11) $$\frac{\partial \mathrm{RH}}{\partial T_{w}}=\frac{1}{P_{ws}\left(T_{d}\right)}\left\{\frac{\partial P_{ws}\left(T_{w}\right)}{\partial T_{w}}-2\left(C_{2}T_{w}+C_{1}\right)\left(T_{d}-T_{w}\right)+C_{2}{T_{w}}^{2}+C_{3}T_{d}+C_{1}T_{w}+C_{0}\right\}$$
  (A12) $$\begin{array}{r} \frac{\partial \mathrm{RH}}{\partial T_{d}}=\frac{1}{{\left[P_{ws}\left(T_{d}\right)\right]}^{2}}\{-[C_{3}\left(T_{d}-T_{w}\right)+\left(C_{0}+C_{1}T_{w}+C_{2}{T_{w}}^{2}+C_{3}T_{d}\right)p_{ws}\left(T_{d}\right)]\;\\ -\frac{\partial P_{ws}\left(T_{d}\right)}{\partial T_{d}}[p_{ws}\left(T_{w}\right)-\left(C_{0}+C_{1}T_{w}+C_{2}{T_{w}}^{2}+C_{3}T_{d}\right)\left(T_{d}-T_{w}\right)]\} \end{array}$$
