# Supplementary material for: Uncertainty Analysis in Humidity Measurements by the Psychrometer Method
Source: Sensors (Basel). 2017 Feb 14;17(2):368. doi: 10.3390/s17020368 (PMC5335993; doi:10.3390/s17020368)
Supplement: Supplementary file 1 [file sensors-17-00368-s001.pdf]

Supplementary Materials: Uncertainty Analysis in Humidity Measurement by the Psychrometer Method

Jiunyuan Chen and Chiachung Chen

Supplement figures legends

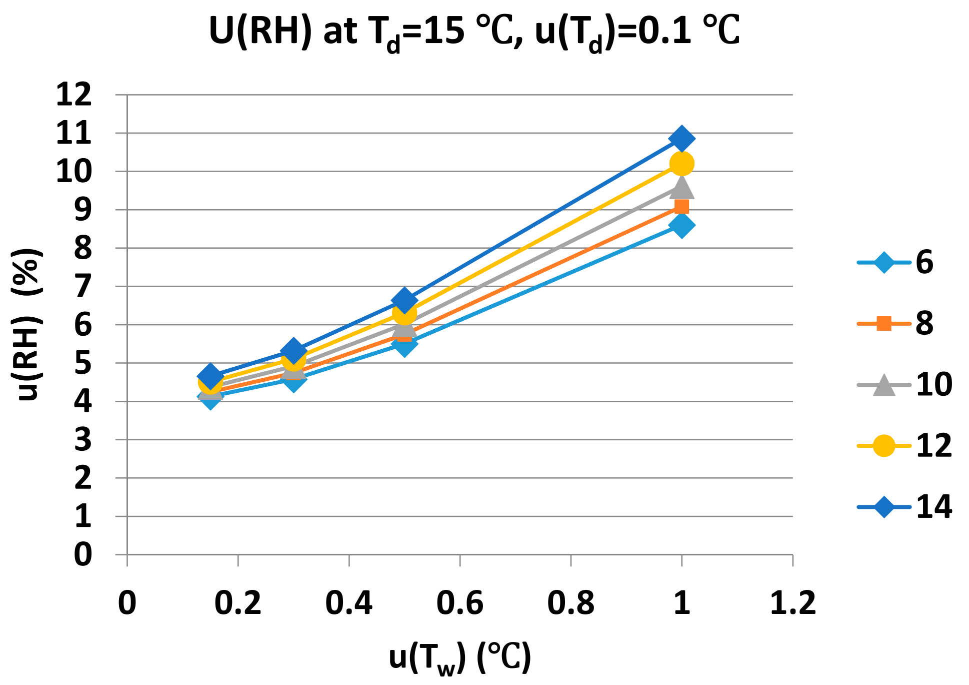

Figure S1. Uncertainties of relative humidity calculated with Equation (15) at T_d = 15 °C, $u(T_d)$ = 0.10 °C, T_w = 6~14 °C and $u(T_w)$ = 0.1~1 °C.

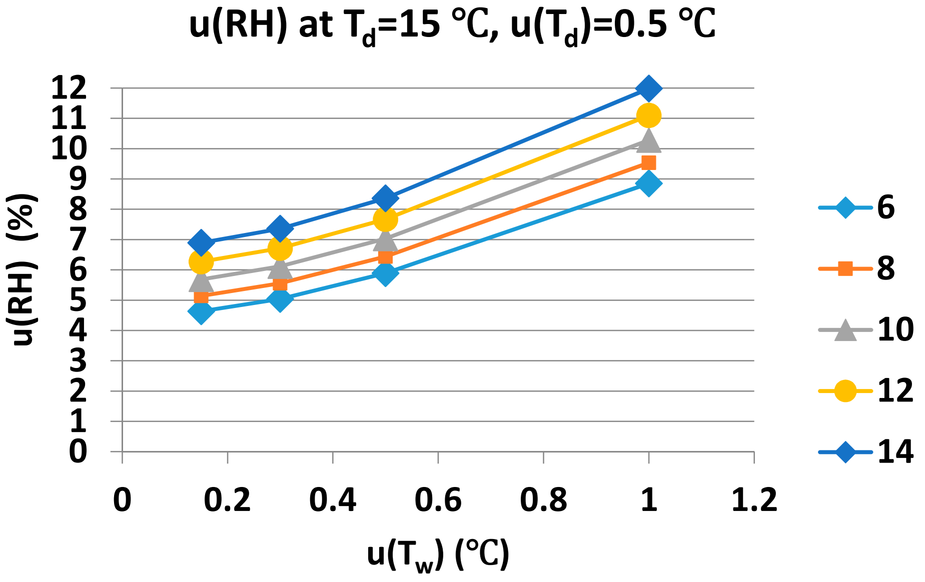

Figure S2. Uncertainties of relative humidity calculated with Equation (15) at T_d = 15 °C, $u(T_d)$ = 0.50 °C, T_w = 6~14 °C and $u(T_w)$ = 0.1~1 °C.

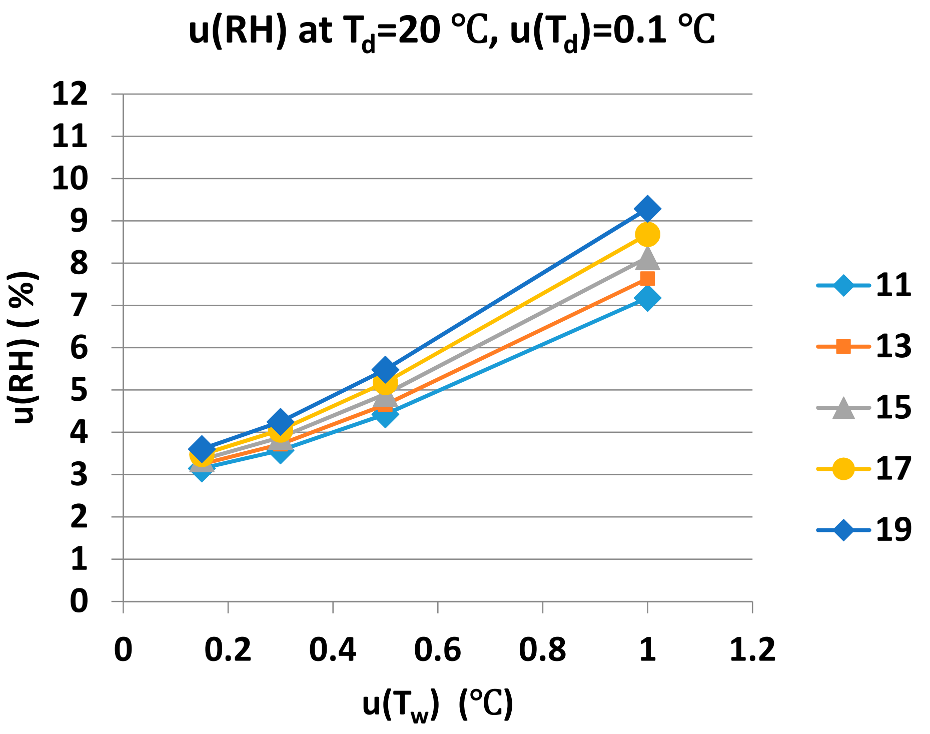

Figure S3. Uncertainties of relative humidity calculated with Equation (15) at T_d = 20 °C, $u(T_d)$ = 0.10 °C, T_w = 11~19 °C and $u(T_w)$ = 0.1~1 °C.

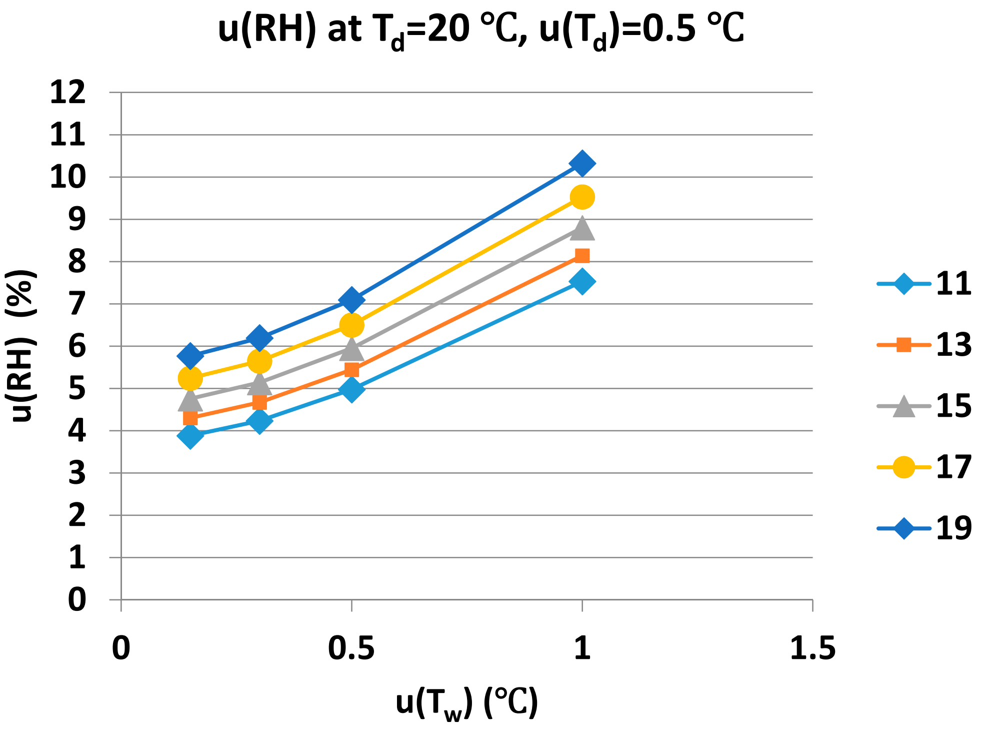

Figure S4. Uncertainties of relative humidity calculated with Equation (15) at T_d = 20 °C, $u(T_d)$ = 0.50 °C, T_w = 11~19 °C and $u(T_w)$ = 0.1~1 °C.

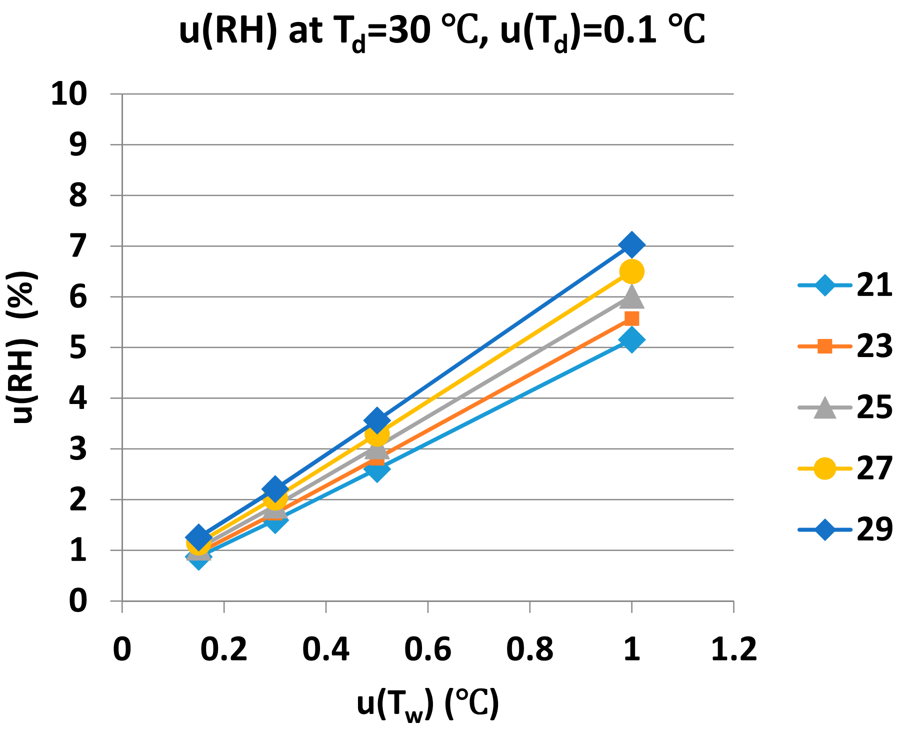

Figure S5. Uncertainties of relative humidity calculated with Equation (16) at T_d = 30 °C, $u(T_d)$ = 0.10 °C, T_w = 21~29 °C and $u(T_w)$ = 0.1~1 °C.

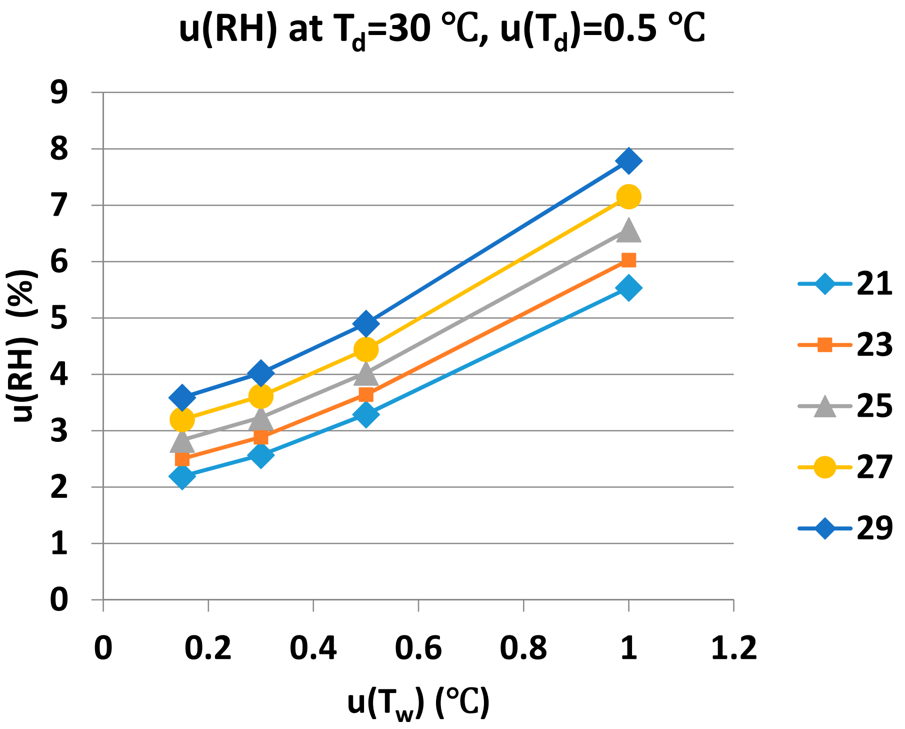

Figure S6. Uncertainties of relative humidity calculated with Equation (16) at T_d = 30 °C, $u(T_d)$ = 0.50 °C, T_w = 21~29 °C and $u(T_w)$ = 0.1~1 °C.

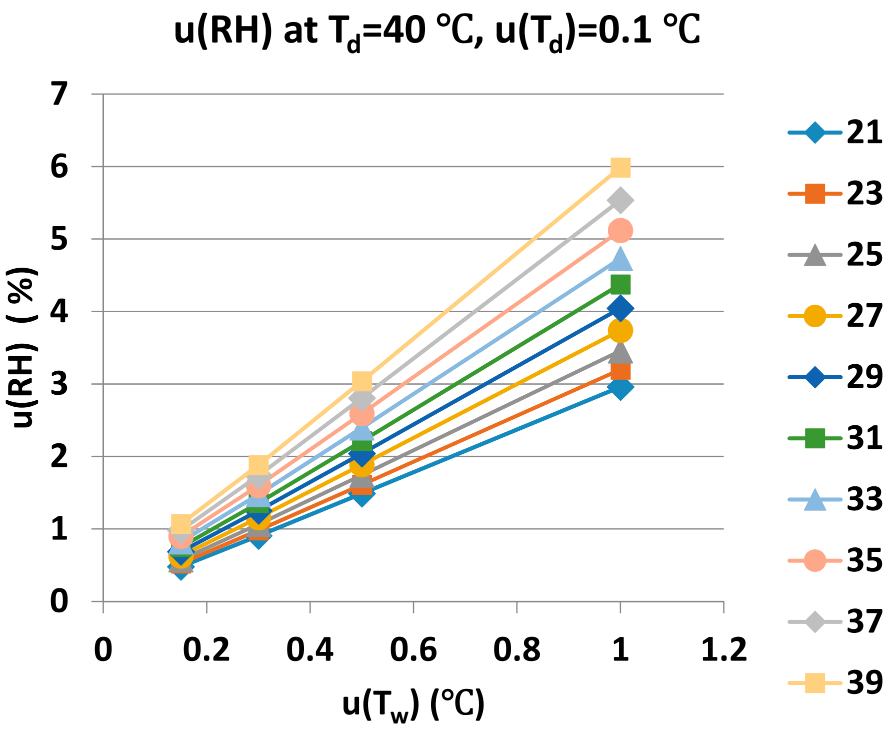

Figure S7. Uncertainties of relative humidity calculated with Equation (16) at T_d = 40 °C, $u(T_d)$ = 0.10 °C, T_w = 21~39 °C and $u(T_w)$ = 0.1~1 °C.

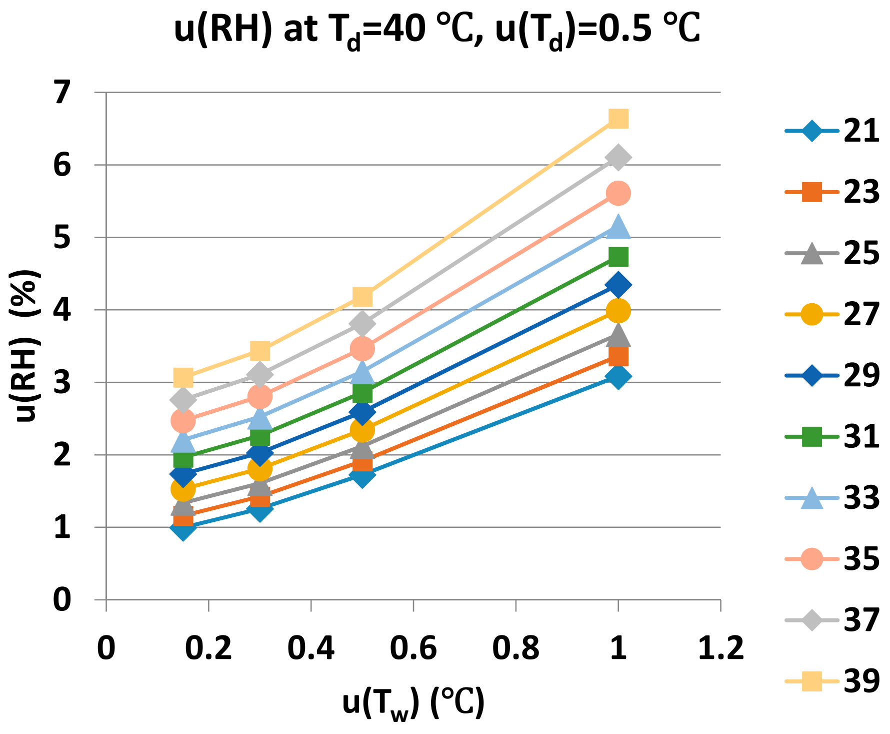

Figure S8. Uncertainties of relative humidity calculated with Equation (16) at T_d = 40 °C, $u(T_d)$ = 0.50 °C, T_w = 21~39 °C and $u(T_w)$ = 0.1~1 °C.
